# Cell Death Mechanisms Induced by Photo-Oxidation Studied at the Cell Scale in the Yeast *Saccharomyces cerevisiae*

**DOI:** 10.3389/fmicb.2018.02640

**Published:** 2018-11-05

**Authors:** Cédric Grangeteau, Florine Lepinois, Pascale Winckler, Jean-Marie Perrier-Cornet, Sebastien Dupont, Laurent Beney

**Affiliations:** Univ. Bourgogne Franche-Comté, AgroSup Dijon, PAM UMR A 02.102, Dijon, France

**Keywords:** photo-oxidation, porphyrins, yeast, two-photon, High power light, blue light

## Abstract

Blue light (400–430 nm) is known to induce lethal effects in some species of fungi by photo-oxidation caused by the excitation of porphyrins but the mechanisms involved remain poorly understood. In this work, we exposed the yeast *Saccharomyces cerevisiae* to a high density light flux with two-photon excitation (830 nm equivalent to a one-photon excitation around 415 nm) and used quasi real-time visualization with confocal microscopy to study the initiation and dynamics of photo-oxidation in subcellular structures. Our results show that the oxidation generated by light treatments led to the permeabilization of the plasma membrane accompanied by the sudden expulsion of the cellular content, corresponding to cell death by necrosis. Moreover, excitation in the plasma membrane led to very fast oxidation and membrane permeabilization (<60 s) while excitation at the center of the cell did not induce permeabilization even after a period exceeding 600 s. Finally, our study shows that the relationship between the laser power used for two-photon excitation and the time required to permeabilize the plasma membrane was not linear. Thus, the higher the power used, the lower the energy required to permeabilize the plasma membrane. We conclude that fungal destruction can be generated very quickly using a high density light flux. Better knowledge of the intracellular processes and the conditions necessary to induce necrosis should make it possible in the future to improve the efficiency of antimicrobial strategies photo-oxidation-based.

## Introduction

Light exerts significant effects on biological cells and is one of the environmental parameters that can be used to manage cellular activities and viability (Fuller et al., 2015). Consequently, photo-oxidation reactions have attracted interest for more than a century with the discovery of the lethal effect of light on microorganisms in the presence of oxygen and photosensitizers (Raab, 1900). In non-photosynthetic microorganisms, singlet oxygen production is linked to cellular cofactors such as flavins, rhodopsins, quinones, and porphyrins that serve as photosensitizers. Photo-oxidative stress is thought to involve singlet oxygen (^1^O_2_) as a primary agent (Redmond and Kochevar, 2006) responsible for cellular damage (Böcking et al., 2000) due to reactions with several cellular macromolecules including proteins, lipids, DNA, and RNA. After the initial action of singlet oxygen, further reactive substances including organic peroxides and sulfoxides are formed. The whole mechanism can lead to cell death and has led to the development of several therapeutic strategies used in oncology (Traitcheva et al., 2003) and for the treatment of various bacterial (Buchovec et al., 2009; Kumar et al., 2015) and fungal infections (Dovigo et al., 2011). The efficiency of the photo-oxidation has mainly been observed for a blue-light (400–430 nm) (Gwynne and Gallagher, 2018). This range of wavelength is used to excite porphyrin, an endogenous photosensitizer, resulting in the production of singlet oxygen. The mechanisms leading to cell death are still not fully understood although certain studies have shown for bacteria that cell death is related to membrane damage (McKenzie et al., 2016; Biener et al., 2017) and others have shown that it is due to the oxidation of DNA (Kim and Yuk, 2017; Yoshida et al., 2017) or genetic changes without damage to membrane integrity (Kim and Yuk, 2017). Thus, several different mechanisms can cause cell death by photo-oxidation and the damage observed could be a result of a combination of these mechanisms. As pointed out recently in their review, Tomb et al. (2018) stated that no conclusive answer has been provided up to now and further works are needed to fully understand the inactivation mechanisms involved. Furthermore, these mechanisms could maybe depend on the microbial species, on exposure times and the intensity of treatments and on oxidation initiation intracellular site. For some of these mechanisms, involving genetic changes in particular, resistances could appear. Short treatments at high power designed in particular to permeabilize the membrane could prevent this resistance. Until now, the results obtained with pathogenic bacteria showing a difference in efficacy between long treatment with weak light intensity and short treatment with high light intensity are contradictory (Murdoch et al., 2012; Barneck et al., 2016). Indeed, Barneck et al. reported that the log_10_-CFU reduction was related to the total dose of light and not its intensity whereas Murdoch et al. showed that for a total dose of equivalent light, a long exposure at low intensity exhibited a greater effect than a short exposure at high intensity.

Our study was conducted in order to investigate the mechanism favored by very high power flux density attainable by two-photon excitation, and to study the effects of varying light power on the time necessary to induce cellular death. Our experimental strategy was based on the direct visualization in quasi real-time of the initiation and dynamics of photo-oxidation on subcellular structures. To do this, cells of the model yeast *Saccharomyces cerevisiae* were exposed to a high density light flux with two-photon excitation under a confocal microscope. Yeasts were labeled with Singlet Oxygen Sensor Green (SOSG) (Flors et al., 2006) and aminophenyl fluorescein (APF) (Price et al., 2009). The use of a two-photon microscope enabled us to target specific part of the cell. This approach allowed us to locate the site of initiation of high power photo-oxidation in cellular plasma membranes and show a non-linear relation between the power of the treatment and the time necessary to induce cell death.

## Materials and methods

### Yeast strains and growth conditions

The *S. cerevisiae* strain BY4742 WT (EUROSCARF, Frankfurt, Germany) was used in this study. The culture was performed on Yeast extract (10 g/L)-Peptone (20 g/L)-Dextrose (20 g/L) medium (YPD) at 25°C in an incubator in the dark to allow growth to the early stationary phase.

### Preparation of the samples for two-photon microscopy

The samples were centrifuged, rinsed twice and put in PBS (Phosphate Buffer Saline). The cell concentration was adjusted to 3.10^7^ cells/mL after which the yeasts were labeled by the cell non-permeable probe SOSG at 6 mg/L. SOSG is a detection reagent that is higly selective for singlet oxygen (Flors et al., 2006). It's a molecule composed of fluorescein and anthracene moieties. In this form, this molecule does not fluoresce even if it is excited. However, in the presence of ^1^O_2_, anthracene moiety reacts with ^1^O_2_ to form endoperoxide. The molecule thus formed will then fluoresce when excited (Kim et al., 2013). The cell-permeable APF probe is O-dearyled upon reaction with ROS to yield strongly fluorescent fluorescein (Setsukinai et al., 2003). APF probe (4 mg/L) was used with 0.1% dimethylsulfoxide (DMSO) to quench the fluorescence due to the hydroxyl radical. This protocol allows to monitor exclusively the formation of ^1^O_2_ (Price et al., 2009). This probe was added 30 min before observation to allow the penetration of the probe in cells. These two probes emit green fluorescence in the presence of ^1^O_2_. Both of these labels are non-fluorescent until reaction with ^1^O_2_.

### Two-photon microscopy observation and photo-oxidative treatment

Yeast cell observations were performed under a Nikon A1-MP scanning two-photon microscope (Nikon, Japan) with a x60 Apo infrared (IR) objective (NA: 1.27, Water Immersion, Nikon, Japan). Two optical band-pass filters (FF01-492/SP-25, FF03-525/50-25, Semrock) with 100 and 50 nm bandwidths, respectively and centers at 445 and 525 nm were used to collect the fluorescence. Excitation was provided by an Coherent Chameleon Vision II mode-locked femtosecond Ti:sapphire laser (140 fs pulse duration, 80 MHz) at 830 nm. Excitation occurs when the absorbed photon energy matches the energy gap between the ground and excited states of molecules of interest. This transition can be achieved by a one photon or a two-photon process. In the latter case, two less energetic photons are simultaneously absorbed. Thus, the high light flux density generated at 830 nm allows a two-photon excitation of molecules usually excited around 415 nm by one photon. The treatment used a cycle with an excitation phase of 2 s in which one cell is exposed to a different mean surfacic light power (1.5.10^4^; 1.7.10^4^; 1.9.10^4^; 2.3.10^4^ and 2.6.10^4^ W/cm^2^ obtained, respectively thanks to 8.6.10^12^; 9.7.10^12^; 1.1.10^13^; 1.3.10^13^; and 1.5.10^13^ W peak power) and an acquisition phase of 4 s on a surface of 1,764 μm^2^ delivering a light power of 400 W/cm^2^ (Figure S1). This cycle was repeated until the cell was permeabilized (Video 1). It should be noted that two-photon absorption phenomenon occurs with a very low statistical probability compare to one than single-photon absorption (Padmanabhan et al., 2010). Furthermore, the thickness of the excited volume is very small (about 0.3 μm). Thus, only a very small part of the emitted power is absorbed and Schönle and Hell (1998) have shown that even powers 10–100 times higher than that used in the present study induce only a weak heating at the focal point.

## Results

**Figure 1:**
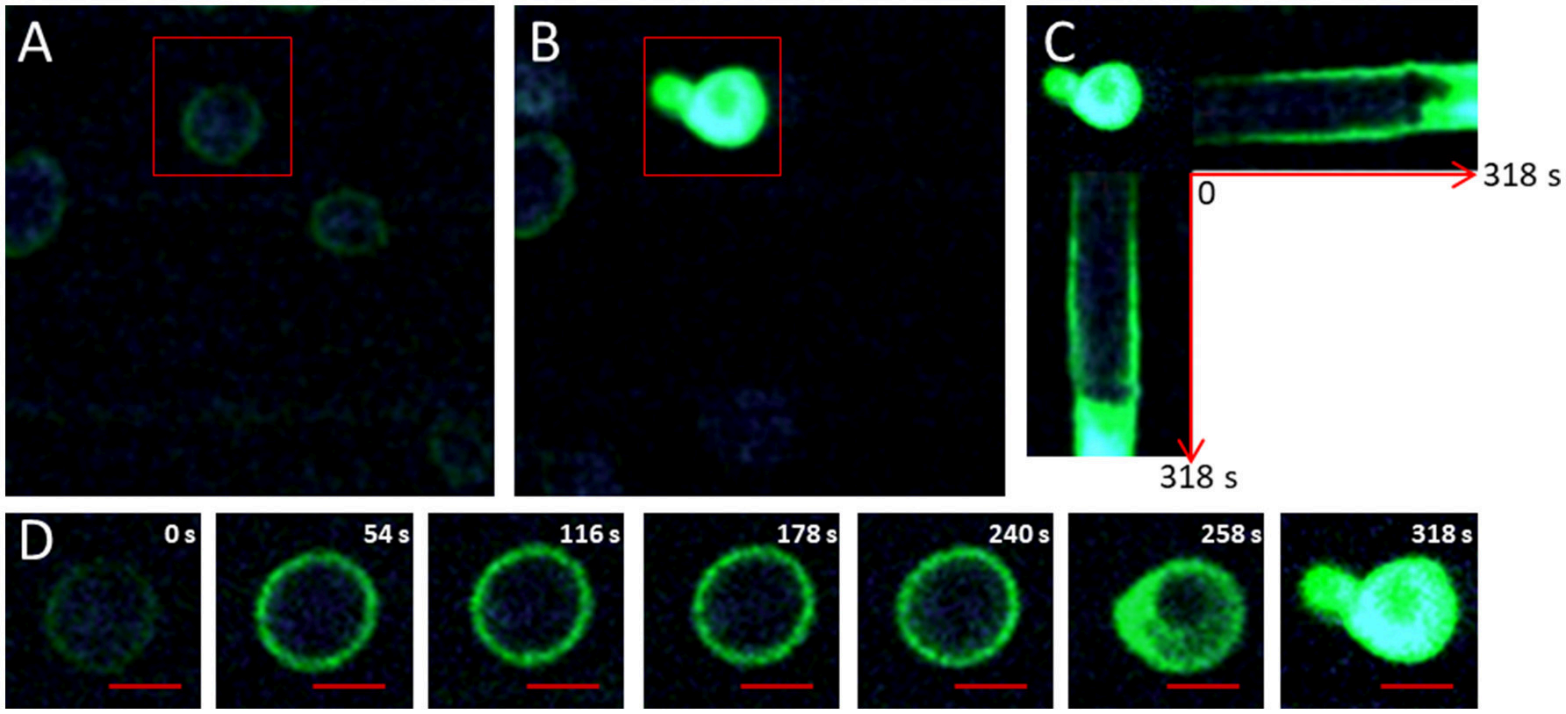
Visualization of ^1^O_2_ production using SOSG. **(A)** Photography of the initial photo-oxidation treatment induced by the production of ^1^O_2_ and labeled by SOSG (6 mg/L) in *Saccharomyces cerevisiae* cells acquired by fluorescence and two-photon microscopy at 830 nm for a light power of 400 W/cm^2^. The red rectangle indicates the targeted cell exposed to light stimulation phases of 1.5.10^4^ W/cm^2^. **(B)** Photograph of the end of the photo-oxidation treatment at t _= 318s_. **(C)** Horizontal and vertical sections of the temporal evolution of fluorescence in targeted cells during the photo-oxidation treatment. **(D)** Crop on the targeted cell exposed to a light power of 1.5.10^4^ W/cm^2^ at 830 nm. The red bar represents 2 μm.

The production of ^1^O_2_ outside the plasma membrane generated by excitation under a two-photon microscope was monitored by the fluorescence of a non-permeant membrane probe SOSG (Figure 1). Initial acquisition before photo-oxidation stimulation showed a fluorescent green ring at the edge of the cell (Figure 1A). This green ring refers to the SOSG probe which emits green fluorescence when the production of ^1^O_2_ starts. It indicates that even during the short acquisition time, the production of ^1^O_2_ started at a light power of 400 W/cm^2^. Green fluorescence became more intense for all cells between the beginning (Figure 1A) and the end of the treatment (Figure 1B), even for those that were not targeted during the stimulation phases. This indicates that the acquisition phase also contributed to the production of ^1^O_2_, but to a much lesser extent than the stimulation phase which led to the total green fluorescence of the cell (Figures 1C,D).

Figures 1C,D show the evolution of fluorescence during the photo-oxidation treatment for a cell targeted with a light power of 1.5.10^4^ W/cm^2^ for 318 s. SOSG quickly became more fluorescent, corresponding to larger production of ^1^O_2_. Between 54 and 240 s, the diffusion and the fluorescence of SOSG seemed to remain the same as before (Figure 1D). At 258 s, fluorescence was observed inside the cell (Figure 1D). The progression of fluorescence in the cell was very fast (Figure 1C) and indicated the permeabilization of the plasma membrane. At 318 s, green fluorescence was emitted out of the cell, indicating the expulsion of cellular content. It should be noted that if a zone containing no any cell is excited, no change in fluorescence indicating a production of singlet oxygen is detected (Figure S2). Due to its non-permeant membrane property, this probe allows visualizing the moment when the membrane is permeabilized. However, the site of production and intracellular distribution of ^1^O_2_ were still unknown.

**Figure 2:**
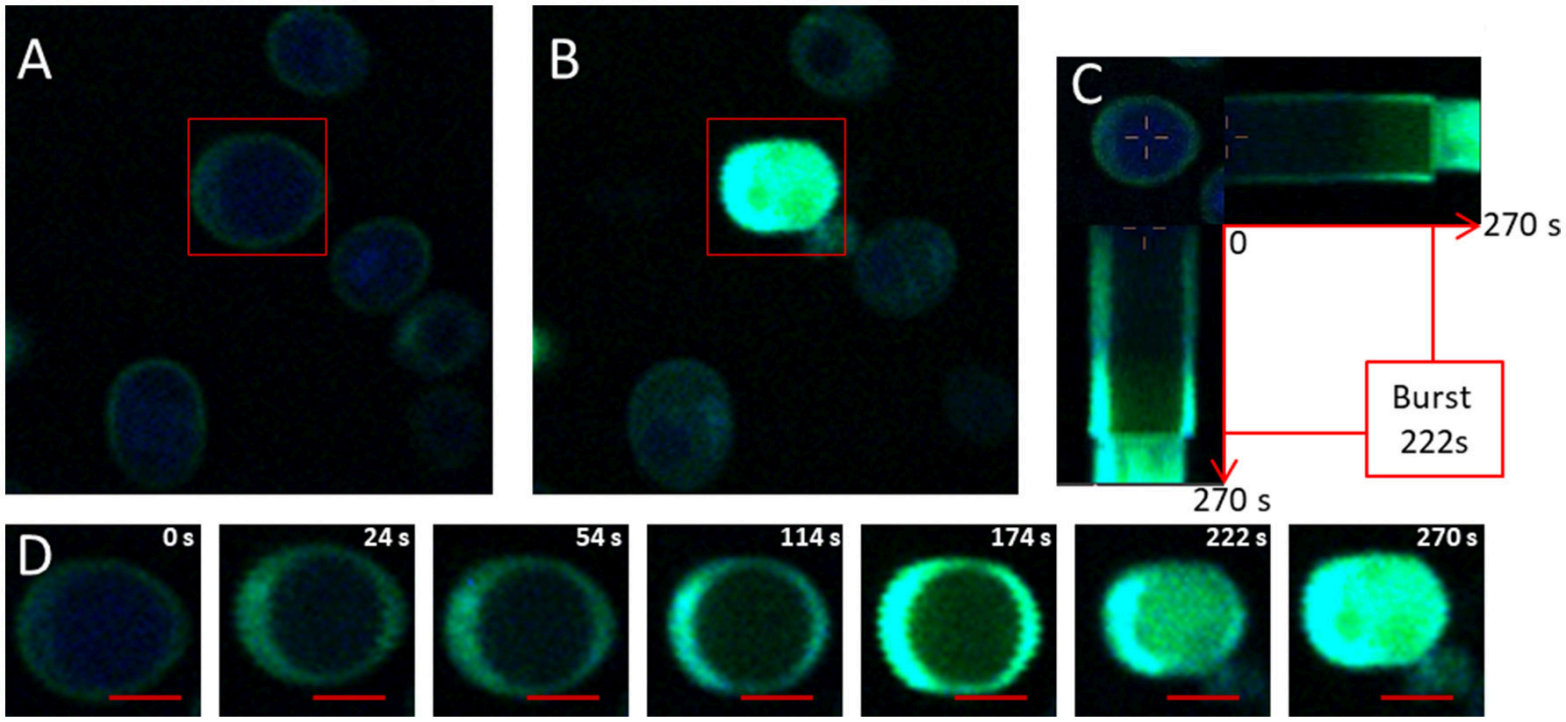
Visualization of ^1^O_2_ production using APF. **(A)** Photograph of the initial photo-oxidation treatment induced by the production of ^1^O_2_ and labeled by APF (4 mg/L) in *Saccharomyces cerevisiae* cells acquired by fluorescence and two-photon microscopy at 830 nm for a light power of 400 W/cm^2^. The red rectangle indicates the targeted cell exposed to light stimulation phases of 1.5.10^4^ W/cm^2^. **(B)** Photograph of the end of the photo-oxidation treatment at t _= 270s_. **(C)** Horizontal and vertical sections of the temporal evolution of fluorescence in targeted cells during the photo-oxidation treatment. **(D)** Crop on the targeted cell exposed to a light power of 1.5.10^4^ W/cm^2^ at 830 nm. The red bar represents 2 μm.

The production of ^1^O_2_ inside the cell following excitation under a two-photon microscope was therefore followed by APF (Figure 2). Figure 2A refers to the first step of acquisition. As for SOSG labeled cells, a green ring was present at the edge (Figure 2A). Since the APF probe is cell permeable, the localization of the fluorescence close to the plasma membrane indicated that it was the primary production site for ^1^O_2_. The red rectangle indicates the targeted zone which corresponds to one *S. cerevisiae* cell. For the target cell, the entire cell appears in green at the end of the treatment. For all the other cells that were not targeted in the stimulation phases, the fluorescence zone was more extended toward the inside of the cell at the end of treatment. This fluorescent zone appeared to occupy the entire intracellular space with the exception of the vacuole, showing that the acquisition phase also contributed to the production of ^1^O_2_, but to a much lesser extent than the stimulation phase.

Figures 2C,D shows the evolution of APF fluorescence during photo-oxidation treatment for a targeted cell with a light power of 1.5.10^4^ W/cm^2^ for 270 s. Fluorescence could be seen clearly (but at low intensity) throughout the cell, except for the vacuole which occupied a large part of the cell volume. The green fluorescence increased with time, which can be explained by the continuous production of ^1^O_2_. At 222 s, 2 phenomena were observed: the volume of the cell decreased suddenly and green fluorescence was emitted out of the cell (Figures 2C,D). These 2 events led us to assume that a breach of the membrane cell occurred. This dual phenomenon could be described as a kind of “photo-oxidative burst,” leading to the disruption of the cellular membrane and thus to the expulsion of the cellular content.

**Figure 3:**
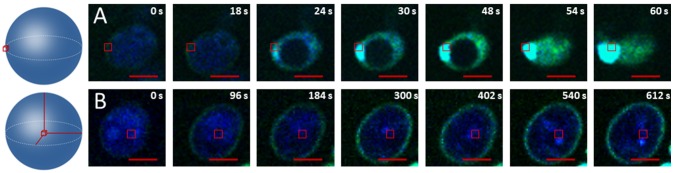
Visualization of ^1^O_2_ production using APF. **(A)** Crop on one cell exposed to a light power of 1.5.10^4^ W/cm^2^ at 830 nm in plasma membrane area. **(B)** Crop on one cell exposed to a light power of 1.5.10^4^ W/cm^2^ at 830 nm in the center of the cell. The red bar represents 2 Wm.

To check that the membrane is indeed the main target of this oxidation phenomenon we compared the oxidation kinetics of cells where the excitation zone was at the center of the cell and of cells where the excitation zone was focused on the plasma membrane area (Figure 3). Figure 3A shows that when the excitation targeted the membrane area, the oxidation kinetics was very fast (<60 s for the permeabilization) and the rupture of the membrane (burst) occurred at the target location. This burst in the targeted area was systematic (Figure S3). In contrast, when targeting the center of the cell, oxidation always started in the membrane and was very slow: after 612 s, the fluorescence of the APF probe was visible only at the level of the cellular periphery and no permeabilization was observed (Figure 3B).

**Figure 4:**
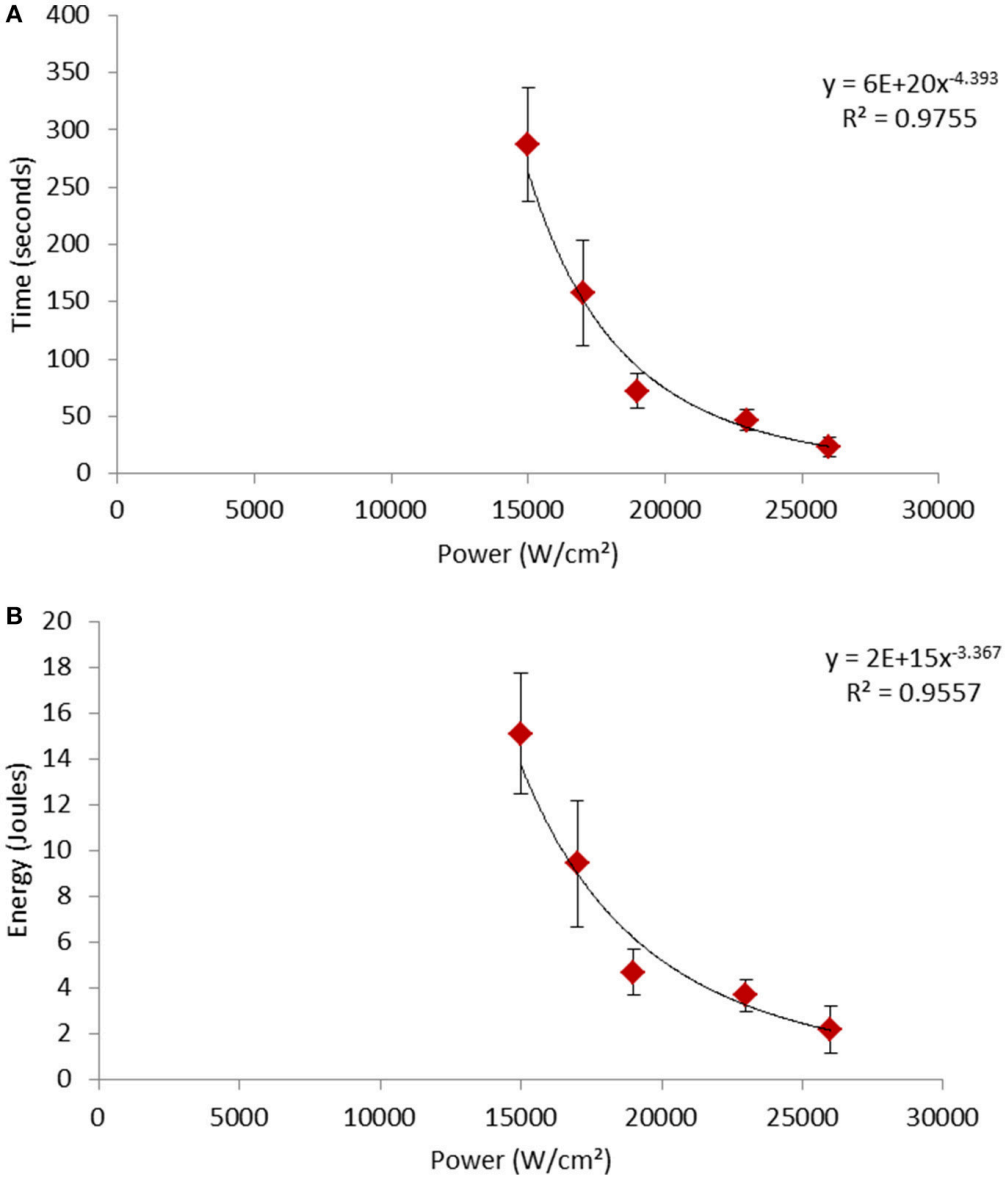
Evolution of **(A)** time and **(B)** energy required to induce the permeabilization of the cells according to the power of the treatment. The black line represents the calculated curve fitting.

We also studied the effect of varying the excitation power on the dynamics of the photo-oxidation phenomenon (Figure 4). Figure 4A shows that the evolution of the time required for cell permeabilization was not proportional to the illumination power. Indeed, the equation of the curve shows that the permeabilization time evolved approximately according to the inverse of the biquadratic number of power [Permeabilization time (s) = 6.10^20^ /Power^4.393^ (W/cm^2^)]. Compared to the time needed for permeabilization with a power of 1.5.10^4^ W/cm^2^, the time for permeabilization with a power of 2.6.10^4^ W/cm^2^ was divided by 12. This non-linear decrease in time led to a non-linear reduction [Energy (J) = 2.10^15^/ Power^3.367^ (W/cm^2^)] of the required energy to permeabilize the cell when increasing the processing power (Figure 4B).

## Discussion

Up to now the elimination of fungi by light treatment has been studied with single-photon excitation (see Wang et al., 2017 for a review). These studies made it possible to demonstrate the possibility of inhibiting growth or even inactivating certain fungi by light treatment without using exogenous photosensitizers (Murdoch et al., 2013; Moorhead et al., 2016; Guffey et al., 2017; Trzaska et al., 2017) and to identify certain endogenous photosensitizers such as porphyrins (Fraikin et al., 1996). However, little is known of the initiation site of oxidation, the intra-cellular dynamics of dispersion and the effect of power excitation modulation with two-photon excitation.

Our results show that the initial fluorescence for both probes formed a ring on the periphery of the cells (Figures 1, 2). Given that the APF is cell-permeable and was therefore located inside and outside the cell whereas the SOSG is not cell-permeable and was thus located only outside the cell, the observation of a fluorescent ring indicated that oxidation was initiated at the plasma membrane. Moreover, in two-photon microscopy the excitation volume is less than one femtoliter (Xu et al., 1996). It is therefore possible to target a particular zone of the cell without exciting the rest of it (Pedersen et al., 2010). Our results show that targeting only the cellular periphery resulted in extremely rapid singlet oxygen production in the targeted area (Figure 3A). This led to very rapid permeabilization in the area of the membrane targeted. When the center of the cells was targeted, the production of ^1^O_2_ was much slower but was still observable at the cellular periphery (Figure 3B). This slow singlet oxygen production at the periphery of the cell was probably related to the energy provided on the whole cell during the image acquisition phase. These results confirm the hypothesis of Fraikin et al. (1996) on the role of a membrane-bound porphyrin-type compound rather than mitochondrial porphyrins in the generation of ^1^O_2_. Our results show the diffusion of ^1^O_2_ in the intracellular space that can be visualized by the APF (Figure 2D). The fluorescence of the APF occupied the whole of the cellular space except the vacuole. DNA alterations may occur at this time due to the accumulation of ^1^O_2_ in the cells. In fact, ^1^O_2_ is known to react with DNA, preferentially guanine (Cadet and Teoule, 1978), resulting in the obstruction of DNA replication (Ribeiro et al., 1992), and mutagenesis (Menck et al., 1993). However, this accumulation and therefore the damage done to the DNA are probably quite limited given the very short lifetime of singlet oxygen: 3.5 μs in water and 14 μs in a lipid solution (Baier et al., 2005). Moreover, given these lifetimes and the diffusion rate, the singlet oxygen diffusion zone in the cytoplasm should be ~200 nm from the membrane (Baier et al., 2005). However, some authors (Grinholc et al., 2015; Kim and Yuk, 2017) showed damage outside the membrane in *Salmonella* and *Staphylococcus aureus* strains and in particular, these authors found DNA oxidation. Firstly, if the diffusion distance of singlet oxygen from the plasma membrane seems too weak to cause damage to the DNA contained in the nucleus in yeast it may be sufficient in bacteria of less than one micron. Secondly, DNA damage could be caused by ROS produced following photoexcitation of photosensitizers. Indeed various ROS can be produced by type I and type II photoreaction. These different ROS are probably responsible for a diversity of cell damage that can synergistically lead to cell death. Regarding the plasma membrane the authors observed damage to the glucose uptake system and a loss of efflux pump activity, but few cells permeabilized. Similarly, Grinholc et al. (2015) observe for *Staphiloccoccus aureus* few permeabilized cells. It is known that photo-oxidation can trigger apoptosis (Oleinick et al., 2002). Thus, cells that have not been permeabilized but have suffered significant damage will continue to be subject to apoptosis even after photo-oxidative treatment has been stopped. This death by apoptosis suggests that a genetic adaptation allowing the cell to resist is possible. Adaptations have already been observed for *S. aureus* following repeated applications of sub-lethal doses (Guffey et al., 2013). In our study, the permeabilization of the plasma membrane was observed by the internalization of the SOSG dye and the expulsion of part of the cellular content (Figure 1). This permeabilization of the membrane with the burst is the ultimate event that can be observed during photo-oxidative treatment and corresponds to cell death by necrosis for which no adaptation is possible. This necrosis could be related to the very high power flux density provided with two-photon excitation and the mechanisms leading to cell death during one-photon excitation with low light flux density could be different. It should be noted that a direct comparison between the powers used in excitation with two photons and the one with one photon is difficult. In fact, the simultaneous absorption of two photons by a receptor molecule occurs with a very low statistical probability (Padmanabhan et al., 2010). Thus, a very large part of the power used in two photons process will not be involved in the excitation.

The leakage of cytosol subsequent to plasma membrane permeabilization could be due to a relaxation of the cell wall. Indeed, the permeabilization of the plasma membrane causes the loss of the turgor pressure exerted on the cell wall. This turgor pressure is related to the osmotic pressure differential between the intra- and extra-cellular compartments and disappears with the permeabilization of the membrane. This results in a release of the cell wall from a stretched state with a decrease in volume and an expulsion of the cellular content (Dupont et al., 2011). Plasma membrane permeabilization was probably due to damage caused by ^1^O_2_. Indeed, it has been shown on plant cells that ^1^O_2_ can cause non-enzymatic peroxidation of the polyunsaturated fatty acids of the membrane (Triantaphylidès et al., 2008). In addition, ^1^O_2_ also reacts with ergosterol (Böcking et al., 2000), the main sterol in *S. cerevisiae* (Dupont et al., 2012) and forms 5α,6α-epoxy-(22E)-ergosta-8,22-dien-3β,7α-diol (8-DED). This oxidation of the lipids (fatty acids and steroids) could lead to a loss of the structuring function of these molecules and thus to the permeabilization of the membrane and then to cell death (Böcking et al., 2000; Gaschler and Stockwell, 2017). Our observations confirm this permeabilization of the plasma membrane caused by photo-oxidation. Lipid oxidation is a chain reaction (Halliwell and Chirico, 1993). Thus, the number of lipids oxidized as a function of time is exponential. Furthermore, it has been shown that in two-photon excitation the number of photons absorbed per unit of time and volume varies quadratically with the incident intensity (Denk et al., 1990; Fischer et al., 1995). These two non-linear phenomena may explain the non-linear relationship between the power and the oxidation of lipids leading to plasma membrane permeabilization, as shown by our results (Figure 4). Moreover, oxidized sterols in the membrane are replaced by unoxidized sterols from the esterified pool (Böcking et al., 2000). The most severe damage to the membrane leading to its permeabilization is therefore caused when the oxidation rate of sterols is much higher than that of their replacement in the membrane. This could explain that the same amount of energy will not have the same effect when applied very quickly as when applied slowly. Therefore, the high power of the focused light implemented by two-photon microscopy offers high destructive efficiency and potential for application. Our results show that using a 10 W laser focused on an area of 100 μm^2^ the effective treatment time would be of the order of 10^−10^seconds/μm^2^. The treatments could therefore be applied by rapid scanning to treat 1 m^2^ in a 100 s. However, the excitation volume in a two-photon process has a thickness of <1 micron. So if the cells are placed on different focal planes as would be the case for a thick sample or a biofilm, each focal plane should be scanned to photo-oxidize all cells. The problem of the thin layer excitation, typical for two-photon microscopy, could be solved by the use of an optical module that generates an axially elongated Bessel focus (Lu et al., 2017).

The present study focusing on initiation sites and the dynamics of photo-oxidation phenomena at the cell scale led to better knowledge of: (i) the areas of the cells that are the most sensitive to photo-oxidation with endogenous porphyrins; (ii) how to apply energy to achieve the greatest destructive efficiency possible. This knowledge could allow increasing the efficiency of photodynamic treatment. Coupled with the benefits of two-photon excitation, it could ultimately provide an effective method of treatment against fungal pathogens.

## Author contributions

SD, CG, and LB designed the experiments, analyzed the data. FL and CG performed the experiments. PW and J-MP-C provided technical support and experimental advice. SD provided a critical review of manuscript. CG and LB wrote the manuscript. All authors read and approved the submitted version.

### Conflict of interest statement

The authors declare that the research was conducted in the absence of any commercial or financial relationships that could be construed as a potential conflict of interest.

## Supplementary material

Figure S1 Cycle with acquisition phase (4 s, 400 W/cm^2^) and excitation phase (2 s) at different light powers (1.5.10^4^; 1.7.10^4^; 1.9.10^4^; 2.3.10^4^ and 2.6.10^4^ W/cm^2^).

Figure S2 Evolution of fluorescence intensity in two excitation area (Region of interest: ROI) delimited by a red rectangle. The two ROI are exposed to light stimulation phases of 1.5.10^4^ W/cm^2^. ROI 1 contains no any yeast cell. ROI 2 contains 1 yeast cell. **(A)** Photography of the initial photo-oxidation treatment induced by the production of ^1^O_2_ and labelled by SOSG (6 mg/L) in *Saccharomyces cerevisiae* cells acquired thanks to fluorescence and two-photon microscopy at 830 nm for a light power of 400 W/cm^2^. **(B)** Photography after 150 s of treatments and **(C)** photography of the end of the photo-oxidation treatment at t = 300 s. **(D)** Evolution curve of fluorescence intensity in Arbitrary Unity (A.U.) for the two ROI.

Figure S3 Visualization of plasma membrane permeabilization for six cells exposed to a light power of 1.5.10^4^ W/cm^2^ at 830 nm in the plasma membrane area.

Video 1 Visualization in video (accelerated 60X) of plasma membrane permeabilization and “photo-oxidative burst” for one cell exposed to a light power of 1.5.10^4^ W/cm^2^ at 830 nm.
