# Supplementary figures and images for: Cell Death Mechanisms Induced by Photo-Oxidation Studied at the Cell Scale in the Yeast Saccharomyces cerevisiae
Source: Front Microbiol. 2018 Nov 5;9:2640. doi: 10.3389/fmicb.2018.02640 (PMC6230929; doi:10.3389/fmicb.2018.02640)

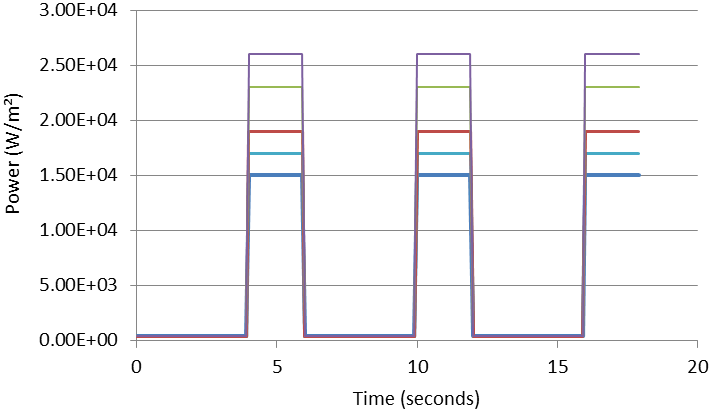

Supplement: Figure S1 — Cycle with acquisition phase (4 s, 400 W/cm2) and excitation phase (2 s) at different light powers (1.5.104; 1.7.104; 1.9.104; 2.3.104 and 2.6.104 W/cm2). [file Image_1.tif]

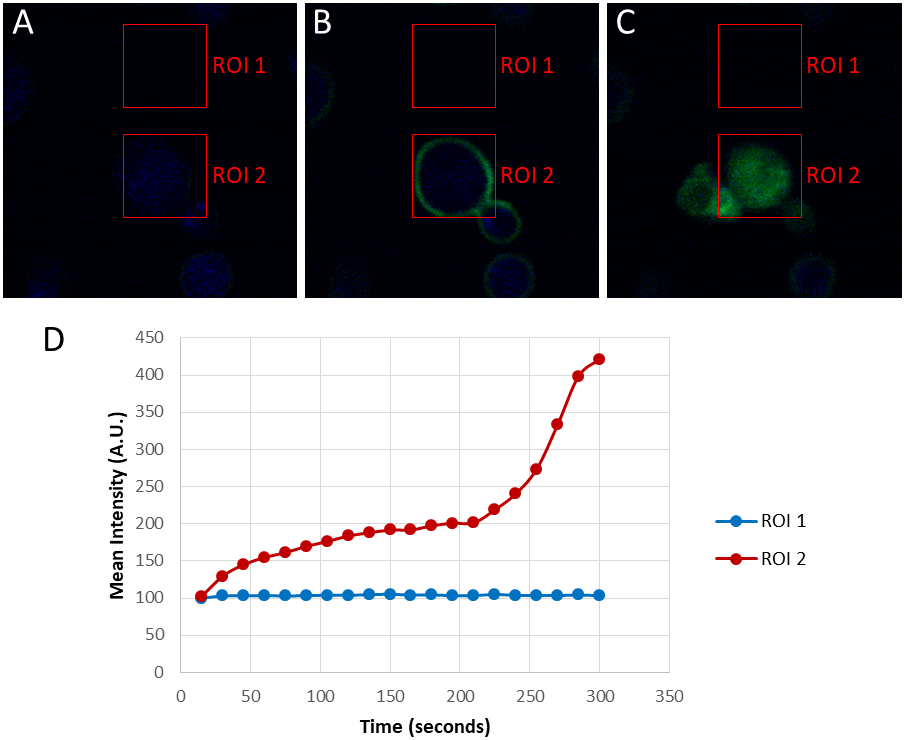

Supplement: Figure S2 — Evolution of fluorescence intensity in two excitation area (Region of interest: ROI) delimited by a red rectangle. The two ROI are exposed to light stimulation phases of 1.5.104 W/cm2. ROI 1 contains no any yeast cell. ROI 2 contains 1 yeast cell. (A) Photography of the initial photo-oxidation treatment induced by the production of 1O2 and labelled by SOSG (6 mg/L) in Saccharomyces cerevisiae cells acquired thanks to fluorescence and two-photon microscopy at 830 nm for a light power of 400 W/cm2. (B) Photography after 150 s of treatments and (C) photography of the end of the photo-oxidation treatment at t = 300 s. (D) Evolution curve of fluorescence intensity in Arbitrary Unity (A.U.) for the two ROI. [file Image_2.tif]

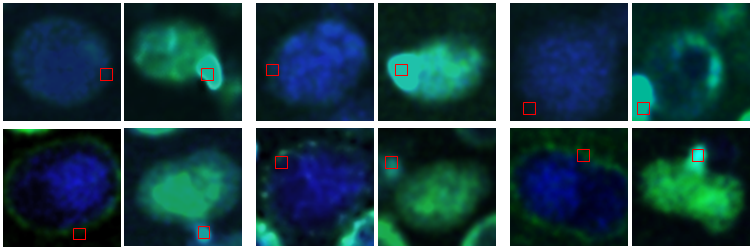

Supplement: Figure S3 — Visualization of plasma membrane permeabilization for six cells exposed to a light power of 1.5.104 W/cm2 at 830 nm in the plasma membrane area. [file Image_3.tif]
